# Intraoral Pleomorphic Adenoma of the Minor Salivary Glands: A Case Series of 10 Cases With Emphasis on Histopathological Features

**DOI:** 10.1155/crid/9151514

**Published:** 2025-01-13

**Authors:** Neetu Jain, Shashi Keshwar, Ashish Shrestha, Mehul Rajesh Jaisani, Iccha Kumar Maharjan, Navin Agrawal

**Affiliations:** ^1^Department of Oral Pathology, College of Dental Surgery, B.P. Koirala Institute of Health Sciences, Dharan, Nepal; ^2^Department of Oral and Maxillofacial Surgery, College of Dental Surgery, B.P. Koirala Institute of Health Sciences, Dharan, Nepal; ^3^Department of Oral Medicine and Radiology, College of Dental Surgery, B.P. Koirala Institute of Health Sciences, Dharan, Nepal; ^4^Department of Conservative Dentistry and Endodontics, College of Dental Surgery, B.P. Koirala Institute of Health Sciences, Dharan, Nepal

**Keywords:** benign mixed tumor, minor salivary glands, pleomorphic adenoma

## Abstract

Pleomorphic adenoma (PA), the most common salivary gland tumor, presents unique challenges due to its diverse clinicopathologic features. The objective of this case series is to highlight the implication of detailed histopathological examination to guide appropriate diagnosis. This study reviews 10 cases of PA diagnosed at B.P. Koirala Institute of Health Sciences, Nepal, between 2011 and 2023. Patients ranged from 16 to 71 years, with a male-to-female ratio of 1:2.3. Most lesions (eight cases) were located on the palate, with additional cases, one on the upper lip and one on the cheek mucosa. Lesion sizes ranged from 1 to 3 cm^2^ and durations from 2 months to 4 years. Clinically, all lesions were well encapsulated, nontender, and nonulcerated and had normal overlying mucosa. Histopathologically, cases included classical PA, myxoid, and cellular types. Common findings were ductal structures filled with eosinophilic material, a “swarm bee” appearance, plasmacytoid cells, and myxoid stroma. Squamous differentiation and psammoma bodies were observed in some cases. No osseous or cartilaginous components were detected. All cases were excised, with no recurrences reported during at least 2 years of follow-up. Hence, proper diagnosis is crucial for effective management and long-term outcomes of PA.

## 1. Introduction

Salivary gland tumors are an extensive range of tumors with diverse clinical, histological, and immunohistochemical characteristics [1]. These tumors make up only 3%–5% of all neoplastic processes in the jaws, making them incredibly rare [2]. Furthermore, regardless of the causative mechanism, salivary gland tumor incidence is generally rising globally [3].

The most frequent type of salivary gland tumor, pleomorphic adenoma (PA), makes up between 40% and 70% of all major and minor salivary gland tumors [4]. PA, first described by Lopes et al., is the most prevalent benign salivary gland neoplasm [4, 5]. PA is defined by the World Health Organization as a localized tumor that presents pleomorphic or mixed characteristics of epithelial origin and is interwoven with mucoid tissue, myxoid tissue, and chondroid masses [6]. Comprising both epithelial and myoepithelial cells organized in different morphological patterns, PA is a benign mixed tumor that is separated from surrounding tissues by a fibrous capsule [7].

The major salivary gland that is most frequently affected is the parotid gland. PAs of the minor salivary glands mostly occur in the soft and hard palate due to greater concentration of salivary glands in these locations and typically presents as a firm or rubbery submucosal mass either without ulceration or surrounding ulceration [8]. The gingiva, cheeks, and lips are uncommon places to find it [9]. Neoplasms of the minor salivary glands, in contrast to the major salivary glands, are rare, representing 10%–15% of all salivary gland tumors [10]. Here, we present a case series of 10 cases on intraoral PA highlighting the implication of detailed histopathological examination to guide appropriate diagnosis.

## 2. Case Presentation

This case series was conducted using archived cases of PA retrieved from the Department of Oral Pathology at B.P. Koirala Institute of Health Sciences, Nepal, covering the period from 2011 to 2023. A total of 10 cases were reviewed. Clinical data, including patient age, sex, lesion location, size, and duration, were compiled. All histopathological slides were analyzed to determine the type of PA and specific features, such as ductal structures and cellular patterns. The clinical information provided for each case was recorded (Table 1). Follow-up data were also reviewed to assess recurrence.

In our study (Table 1), cases are seen with a wide age gap. Half of the cases (five cases) are seen in the middle age group, although cases are also reported at very early ages like 24, 20, and 16 years (juvenile PA); male: female ratio here is 1:2.3. The size of most of the reported lesions are between 1 and 3 cm^2^, with a duration ranging from 2 months to 4 years.

We did not receive any cases of PA in the parotid gland. Most of our reported (Table 1) cases (eight cases) were seen on the palate. We also reported cases of occurrence at some unusual sites, such as one case on the upper lip and one case on the cheek mucosa, where clinically the cases were diagnosed as fibroma and lipoma, respectively.

All the reported cases (Table 1) were well encapsulated, nontender, nonulcerated, and nonfluctuant, with no pain and normal overlying mucosa.

**Figure 1:**
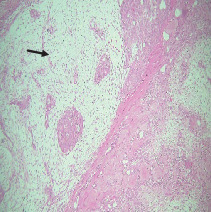
Myxoid stroma.

**Figure 2:**
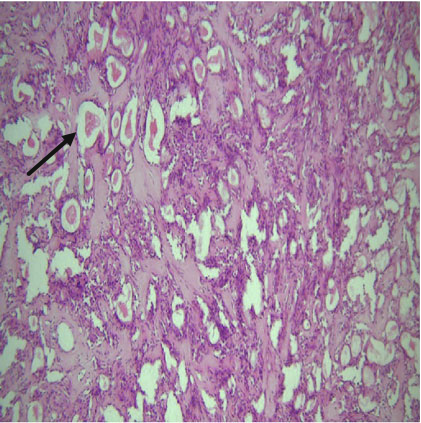
Duct-like structures filled with eosinophilic material in a hyalinized stroma.

**Figure 3:**
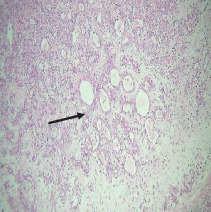
Swarm bee appearance.

**Figure 4:**
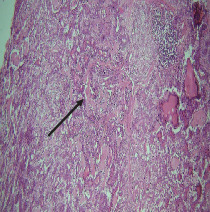
Cords and strand-like arrangement of cells.

**Figure 5:**
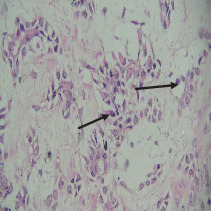
Plasmacytoid cells and mucous cells in a loose fibrillar stroma.

**Figure 6:**
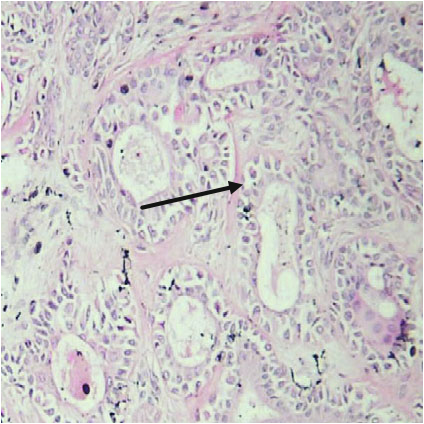
Clear cells lining the ductal structure.

**Figure 7:**
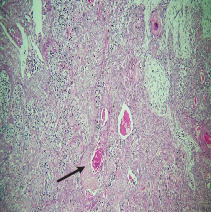
Squamous differentiation and formation of keratin pearl.

**Figure 8:**
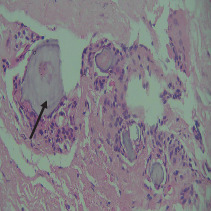
Psammoma bodies within the collagenous stroma.

On histopathological examination (Table 2), half of the cases (five cases) were classical PA, a few seemed to be of the myxoid type (Figure 1), and only one case was cellular. All the cases had ductal structures filled with eosinophilic material (Figure 2) in most of them, with most cases having a swarm bee appearance (Figure 3) of the cells and ducts together, although some areas also revealed a reticular pattern of cellular arrangement (Figure 4). Plasmacytoid cells (Figure 5) were seen in most of the cases, whereas clear cells (Figure 6) were reported only in a few. In addition to the myxoid stroma (Figure 1), hyalinized stroma (Figure 2) was also a common finding. Squamous differentiation was reported only in three cases, with the formation of keratin pearls (Figure 7) in one of them. In addition, psammoma bodies (Figure 8) were evident in one of the cases (Table 3). No osseous or cartilaginous areas are reported in our case series. All the cases were surgically excised, and on follow-up, for at least 2 years, no report of reoccurrence has been found till now.

**Figure 9:**
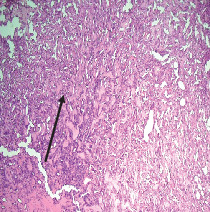
PA resembling schwannoma with numerous spindle cells.

The clinical diagnosis of four reported cases was quite different from the histopathological diagnosis. As in our reported cases, the clinical diagnosis of fibroma, lipoma, and mucoepidermoid carcinoma (MEC) was considered (Table 1). Whereas on histopathological diagnosis, the cases were diagnosed as PA. However, histopathologically, a few cases also resembled schwannoma (Figure 9) and oral squamous cell carcinoma (OSCC) (Figure 7).

## 3. Discussion

The parotid gland is the most common site for PA [6]. However, we did not report any cases that occurred in the parotid gland; this might be because extraoral lesions are not operated on in our dental college. The male-to-female ratio varies from 1:1.4 to 1:1, and according to our findings, it was around 1:2.3 [5, 11]. The prevalence is believed to be common in the fourth and sixth decades of life [5, 11]. Nevertheless, we also reported cases at a very early age of 24, 20, and 16, so cases at such an early age should also be considered to avoid overenlargement and recurrence. The mean size of most mixed intraoral tumors is less than 3.0 cm, which is like our findings (Table 1) [12].

Histologically, it is a benign tumor made up of cells that can differentiate into mesenchymal cells (chondroid, myxoid, and osseous) and epithelial cells (ductal and nonductal) [13]. The ability of tumor cells to differentiate into fibrous, hyalinized, myxoid, chondroid, and osseous areas because of metaplasia or the actual products of tumor cells accounts for its morphologic complexity [5, 6, 14]. Many theories have been put forth to explain the histogenesis of PA. It can be the result of reserve cells in the intercalated duct and myoepithelial cells. The tumor may be caused by neoplastically modified epithelial cells that have the capacity to differentiate in multiple directions [15]. Chromosome 12q 13–15 has been determined to have cytogenetic anomalies [5, 16]. On chromosome 8q12, the putative pleomorphic adenoma gene 1 (PLAG1) has been located [13]. Furthermore, the etiology of the disease has been proposed to involve genetic predisposition, chemical exposure, and tobacco use [17].

Histologically, the coordinated proliferation of luminal and nonluminal (basal/myoepithelial) cells, with the latter tending to predominate numerically over the former, is the only consistent feature. The neoplastic cells of these PA cells are compared to a “swarm of bees” (Figure 3), encasing ductal structures, and this was the common feature in most of our diagnosed cases as well. Therefore, the elements that give rise to this typical histology hold the key to the diagnostic criteria of PA [18]. Wenig et al. defined PA as “admixture of epithelial and myoepithelial cells consisting of gland-like structures, ducts, cell nests, cords, spindle-shaped and plasmacytoid cells, and mesenchyme-like chondromyxoid tissue” [19]. If the glands and ducts account for less than 5% of the histology, the condition is classified as myoepithelioma. The proliferation of duct-like units made up of luminal cells and one to many layers of nonluminal cells with few or no myxoid or chondroid elements results in cellular versions. The amount of chondroid matrix and the degree of complete differentiation of tumor cells into chondrocytes vary. Osteoid matrix and osseous differentiation are rarely seen. We also did not appreciate any chondroid or osseous matrix [18]. These cells, which are often spindle-shaped or epithelioid, are organized in narrow, sinuous, and anastomosing cords that produce a reticular pattern in some regions. Hyaline to myxoid (Figures 2 and 1) materials may be prominent between nonluminal cells, which was seen in most of our cases as well (Tables 2 and 3).

Hyaline or plasmacytoid cells are major cells in PA, which appear as round to polygonal-shaped nonluminal cells with an increased amount of bright eosinophilic staining cytoplasm, and the nucleus is displaced to one side of the cell. PA of minor salivary gland origin is more likely to contain many plasmacytoid cells. Also, in the case of PA, there can be very little cellular or nuclear pleomorphism, but there are rarely mitoses [18]. Plasmacytoid cells (Figure 5) were a constant finding in most of our cases and were also very helpful in diagnosing cases of PA. In certain tumors, luminal cells exhibit varying degrees of focal differentiation into goblet, sebaceous, or squamous cells. About 25% of PA patients have some squamous metaplasia, which can be diffuse or prominent and show up as cysts filled with keratin. We also reported similar findings. Major squamous metaplasia development seems to be associated with nonluminal tumor cells. The formation of psammoma bodies might also take place [18]. Only one of our cases showed the presence of psammoma bodies (Figure 8) (Table 3).

Although it has distinct histological characteristics, PA can sometimes be misdiagnosed as a malignancy due to its mimicking ability, atypical or metaplastic cytomorphology, and morphological features that resemble those of salivary gland carcinomas that have already been established [20]. These factors make PA detection difficult in several ways. PA can be mistaken for myoepithelioma, MEC, adenoid cystic carcinoma (ACC), basal cell adenoma (BCC), epithelial-myoepithelial carcinoma, schwannoma, myxoma, myxoid malignant fibrocytoma, or OSCC due to its variable histopathological presentation. It is very important to differentiate PA from these lesions due to varying clinical management. In our cases also, there was a resemblance between PA and MEC, OSCC (Figure 7) (Table 1), and Schwannoma (Figure 9). However, to differentiate, a thorough knowledge of differentiating and characteristic features is needed. To differentiate, it is important to note that although PA shares a lot of histopathologic similarities with these lesions, it lacks cellular atypia, increased mitotic activity, or capsular or perineural invasion. The presence of plasmacytoid cells and the swarm bee arrangement pattern of cells also help to differentiate PA from other lesions. In our reported cases, the diagnosis of fibroma, lipoma, MEC, schwannoma (Figure 9), and OSCC (Figure 7) was considered, but on careful histopathological diagnosis, the cases were diagnosed as PA.

The diagnosis of this tumor is challenging due to the variety and intricacy of tumors. Immunohistochemistry (IHC) and molecular analysis are useful adjuncts as a result. The zinc finger transcription factor, encoded by the PLAG1, is typically activated by the chromosomal translocation of 8q12 in the salivary gland subgroup PA [21]. The PLAG1 stain can be used to identify the appearance of the PLAG1 oncogene. The primary benefit of utilizing this specific stain for salivary gland tumors is its ability to primarily identify PA and carcinoma ex-PA. This unique marker can be used to sign out cases of head and neck (H&N) tumors because it has good specificity for detecting PA [22]. Other markers that can be used but are not very specific, are lymphoid enhancer binding factor 1 (LEF1) and NR4A3. With positive predictive values of 95% for PA and 97% for BCC, respectively, a positive LEF1 result indicates a benign tumor; further IHC studies with LEF1 are needed [23].

The proteoglycan (PG) and cartilage link protein (LP), two hyaluronan (HA)-binding molecules, are the structural elements of the hydrated extracellular matrix. Due to the significant functions these chemicals play in the tumor microenvironment, Kuwabara et al. investigated the distribution of versican, aggrecan, HA, and LP in salivary gland tumors by double staining and other histochemical and immunohistochemical techniques. The result showed that the tissues of PA and ACC both contained LP. In the chondromyxoid matrix of PA, LP colocalized with both HA and aggrecan, indicating the existence of a HA–LP–aggrecan complex. They concluded that salivary gland tumor behavior and immunohistochemical examination of these chemicals could serve as a diagnostic auxiliary in cases of PA and ACC [24].

PA usually manifests as a slow-growing or painless nodule that patients often do not notice [25, 26]. Delay in medical attention can cause the tumor to grow larger, increasing the difficulty of surgical removal and the possibility of recurrence. Patient age and tumor size are the two clinical characteristics that have been linked to a higher risk of recurrence to date, and this finding is consistent with most findings in the literature [27, 28]. Sometimes malignant transformation into carcinoma ex-PA occurs in PA, and the tumor may spread. PAs can metastasize less frequently without undergoing a histologically malignant transformation, particularly in cases where an excision was performed [29, 30]. PA metastasizes most frequently to the lung, cervical lymph nodes, and bone [31].

Metastatic PA is an uncommon occurrence, typically occurring following incomplete removal of the original tumor, sometimes emerging at a later stage. Interestingly, this type of metastasis does not exhibit the expected cellular abnormalities. Therefore, extended monitoring, particularly in such cases, is essential. Its cause is not fully understood, and its histological appearance, currently indistinguishable from a benign PA, markedly contrasts with its clinically aggressive behavior. Thorough and precise removal of the primary lesion may lower the chances of distant spreading. The emergence of metastases within 10 years of initial diagnosis and the presence of the disease in multiple remote locations significantly diminishes survival rates [30].

Thus, all cases should be carefully histopathologically evaluated after a clinical evaluation followed by IHC as far as possible to avoid any complications, as clinical management generally varies for different lesions. In our institute, all the reported cases were surgically removed (Table 1), which is believed to be the best course of action for PA, and to date, there are no reports of recurrence for the abovementioned cases.

## 4. Conclusion

The recognition of pleomorphic patterns among tumors of salivary glands presents considerable difficulty in diagnosis, particularly to those world practitioners who see it rarely. Multicentric and more extensive histopathological database studies are vital for the advancement of diagnostic power and better treatment of patients with salivary gland tumors. Undertreatment or overtreatment due to misdiagnoses will severely impact the overall treatment of the patient. Our research addresses this issue by explaining the lesions of PA histopathologically to improve the diagnosis and treatment of the disease.

## 5. Limitations

We did not report any cases that occurred in the parotid gland; this might be because extraoral lesions are not operated on in our dental college. It is also important to consider that this is not an epidemiological study and that the sample included is not representative of the population.

## Figures and Tables

**Table 1:** Clinical findings of reported cases.

**SR no.**	**Age**	**Gender**	**Site**	**Size (cm** ^**2**^ **)**	**Duration**	**Clinical diagnosis**	**Treatment**	**Habit and medical history**
1	50	Female	Palate	3 × 3	1 year	Minor salivary gland tumor	Surgical excision	Nothing significant
2	40	Female	Upper lip	1 × 0.5	1 year	Fibroma	Surgical excision	Nothing significant
3	24	Female	Palate	3 × 3	2–3 years	Mucoepidermoid carcinoma (MEC)	Surgical excision	Nothing significant
4	34	Male	Palate	2.5 × 1.5	4 years	PA	Surgical excision	Tobacco chewing
5	71	Male	Palate	1.5 × 2	2 months	Benign salivary gland tumor	Surgical excision	Tobacco chewing, smoking, and alcohol Epilepsy and on medication
6	39	Female	Cheek	1 × 1	2 months	Lipoma	Surgical excision	Nothing significant
7	16	Female	Palate	3 × 3	7 months	PA	Surgical excision	Nothing significant
8	48	Male	Palate	1 × 1	1 year	PA	Surgical excision	Diabetes mellitus with no medication.
9	52	Female	Palate	0.6 × 0.6	1 year	Inflammatory hyperplasia	Surgical excision	Nothing significant
10	20	Female	Palate	3 × 2	1 year	PA	Surgical excision	Nothing significant

**Table 2:** Type of PA based on the predominant features.

**Serial no.**	**Myxoid type**	**Classic type**	**Cellular type**
1	−	−	+
2	+	−	−
3	−	+	−
4	−	+	−
5	+	−	−
6	+	−	−
7	+	−	−
8	−	+	−
9	−	+	−
10	−	+	−

**Table 3:** Histopathologic findings of the cases of PA.

**Cellular component**		**Morphological pattern**		**Stromal component**	
Category	Percentage	Category	Percentage	Category	Percentage
Plasmacytoid cells	90	Ductal	100	Hyalinized	50
		Eosinophilic material within the ductal component	80		
Spindle cells	100	Cystic	30	Myxoid	90
Clear cells	40	Reticular	30	Osseous and chondroid	0
Squamous cells	30	Solid	0	Psammoma body	10
Mucous cells	20	Swarm bee	70	Keratin pearl	10

## Data Availability

The data supporting this article are available from the corresponding author or first author on reasonable request.
